# Dysfunction in the hierarchy of morphometric similarity network in Alzheimer’s disease and its correlation with cognitive performance and gene expression profiles

**DOI:** 10.1017/S0033291725000091

**Published:** 2025-02-12

**Authors:** Chuchu Zheng, Wei Zhao, Zeyu Yang, Shuixia Guo, the Alzheimer’s Disease Neuroimaging Initiative

**Affiliations:** 1 School of Public Health, Shanxi Medical University, Taiyuan, People’s Republic of China; 2 MOE-LCSM, School of Mathematics and Statistics, Hunan Normal University, Changsha, People’s Republic of China; 3 Key Laboratory of Applied Statistics and Data Science, Hunan Normal University, College of Hunan Province, Changsha, People’s Republic of China

**Keywords:** Alzheimer’s disease, gene expression profiles, gradient, morphological similarity network

## Abstract

**Background:**

Previous research has shown abnormal functional network gradients in Alzheimer’s disease (AD). Structural network gradient is capable of capturing continuous changes in brain morphology and has the ability to elucidate the underlying processes of neurodevelopment. However, it remains unclear whether structural network gradients are altered in AD and what associations exist between these changes and cognitive function, and gene expression profiles.

**Methods:**

By constructing an individualized structural network gradient decomposition framework, we calculated the morphological similarity network (MSN) gradients for 404 subjects (186 AD patients and 218 normal controls). We investigated AD-related alterations in MSN gradients, along with the associations between MSN gradients and cognitive function, MSN topological properties, and gene expression profiles.

**Results:**

Our findings indicated that the principal MSN gradient alterations in AD were primarily characterized by an increase in the primary and secondary sensory cortices and a decrease in the association cortex 1. The primary and higher-order cortices exhibited opposite associations with cognition, including executive function, language skills, and memory processes. Moreover, the principal MSN gradients were found to significantly predict cognitive function in AD. The altered gradient pattern was 14.8% attributable to gene expression profiles, and the genes demonstrating the highest correlation are involved in metabolic activity and synaptic signaling.

**Conclusions:**

Our results offered novel insights into the underlying mechanisms of structural brain network impairment in AD patients, enhancing our understanding of the neurobiological processes responsible for impaired cognition in patients with AD, and offering a new dimensional structural biomarker for AD.

## Introduction

Alzheimer’s disease (AD) represents the predominant type of dementia, marked by significant neurodegeneration, alongside symptoms such as memory impairment and cognitive degradation. It is characterized by pathological processes involving β-amyloid deposition and tau pathology (Braak, Alafuzoff, Arzberger, Kretzschmar, & Del Tredici, 2006; Villemagne et al., 2013). However, the neurobiological mechanisms of AD have not yet been elucidated.

The foundational organizational structure of the human brain encompasses a hierarchical architecture, facilitating the encoding and integration of information from sensory perception to cognitive processes (M. Mesulam, 2012; M. M. Mesulam, 1998). This hierarchical architecture serves as an optical organization for information transferring within the human brain (Wang et al., 2024), and uncovering it could provide insight into how the integrated nature of neural processing can give new perspectives on understanding the roots of function and dysfunction (Xue et al., 2023). To study the hierarchical nature of the brain, researchers have proposed the concept of gradients, which are low-dimensional manifold representations of high-dimensional brain network features obtained by diffusion map embedding algorithm (Margulies et al., 2016). Different gradients represent different axes of variance of brain network features, along which cortical regions are arranged in a spatial continuum (Huntenburg, Bazin, & Margulies, 2018; Margulies et al., 2016). The principal gradient explains the maximum variation in the brain network. An increasing array of studies on gradients have sought to examine the hierarchy of brain organization. Notably, the principal functional network gradient delineates a spectrum spanning unimodal (sensory–motor) regions to transmodal (default mode) regions in healthy adults (Huntenburg et al., 2018; Margulies et al., 2016). This spectrum is consistent with the hierarchical architecture of the brain, and many diseases involve abnormal changes in this spectrum (Dong et al., 2023; Hong et al., 2019; Xia et al., 2022). A previous study has found that AD is associated with an increase in functional network gradients in unimodal regions and a decrease in transmodal regions (Zheng, Zhao, Yang, & Guo, 2024b).

In parallel, structural network gradients delineate the spatial continuum in morphology or microstructure, offering a pathway to unveil the underlying mechanisms of neurodevelopment (Burt et al., 2018; Huntenburg et al., 2018). To construct the structural network gradients, we first need to construct the structural network. Traditionally, the construction of the structural covariance network based on structural magnetic resonance imaging (sMRI) has been carried out almost exclusively at the group level, ignoring differences at the individual level (Modinos et al., 2009). A recent study suggests that the structural characteristics of the human cortex can be more accurately assessed by integrating multiple sMRI-derived morphometric indices for each specific region. This could include, for example, combining cortical thickness with gray matter volume (Sabuncu et al., 2016). On this foundation, a morphological similarity network (MSN) was proposed, which combined multiple sMRI-derived cortical characteristics, including cortical thickness (CT), cortical volume (CV), cortical area (CA), gaussian curvature (GC), and mean curvature (MC). MSN quantifies the similarity between cortical regions, and instead of measuring inter-regional correlations between individual cortical morphological features of a group of subjects, it measures the correlation between multiple cortical morphological features of each individual, thus allowing to obtain structural brain networks at the individual level (M. J. Cai et al., 2023; Seidlitz et al., 2018). MSN has a complex topological organization, including modularity. MSN modules recapitulate known classes of cortical cytoarchitecture, and the edges of the MSN strongly correlate with regional gene co-expression in the human brain. In rhesus monkeys, brain regions with higher morphometric similarity were found to be more likely to form axonal tracts with each other (Seidlitz et al., 2018). In addition, MSN could be considered a neuroimaging phenotype correlating structural alterations in the brain with transcriptomic features, thus capturing cellular, molecular, and functional features of the brain. However, MSN itself characterizes the brain from a discrete perspective, while MSN gradient analysis can capture how various brain regions are assembled from a more integrated perspective. Therefore, mapping MSN gradients can reproduce the hierarchical properties of cortical organization (Li et al., 2021; Seidlitz et al., 2020). In healthy subjects, the first MSN gradient manifests in the sensory and motor cortex at both extremes and the first MSN gradient is strongly correlated with cortical organization properties (Yang et al., 2021). In patients with major depression, the first MSN gradient decreases in sensorimotor regions and increases in visual-related regions compared to normal controls (Xue et al., 2023). However, it is still unclear whether and how the first MSN gradient changes in AD.

As a genetically driven disease, AD has a heritability of 50–70 percent (Gatz et al., 1997; Pedersen, Gatz, Berg, & Johansson, 2004). Emerging evidence indicates that genetic factors are crucial in the formation and organization of the human brain connectome (Arnatkeviciute et al., 2021; Thompson et al., 2001). The integration of microarray-based gene expression data from post-mortem adult brains, such as those provided by the Allen Human Brain Atlas (AHBA) (Hawrylycz et al., 2012), with neuroimaging techniques have led to the development of the emerging field of neuroimaging transcriptomics. This approach offers a feasible method for identifying the genetic mechanisms that influence various neuroimaging phenotypes. It also establishes a crucial link between the detailed patterns of gene expression at the micro level and the large-scale organization of brain networks (Fornito, Arnatkevičiūtė, & Fulcher, 2019; F. Liu, Tian, Li, Li, & Zhuo, 2019; J. Liu, Xia, Wang, Liao, & He, 2020; Richiardi et al., 2015; Xia et al., 2022; Xue et al., 2023; Zhu et al., 2021). Specifically, prior work identified a correlation between alterations in functional connectome gradients in patients with AD and gene expression profiles (Zheng, Zhao, et al., 2024b). Therefore, elucidating the association between alterations in MSN gradients and genetic expression in AD will contribute to a deeper comprehension of the molecular genetic underpinnings driving changes in MSN gradients in AD.

To fill these gaps, we applied both the sMRI and gene expression datasets to investigate the first MSN gradient in patients with AD, and we analyzed their correlations with transcriptome profiles and cognitive functions. Specifically, our hypotheses were that: (i) the first MSN gradients alter in AD; (ii) the first MSN gradients in AD are associated with cognition; and (iii) the regions with altered first MSN gradient in AD are related to transcriptome profiles enriched in specific biological function.

## Methods

### Subjects

The dataset employed in our research was obtained from the Alzheimer’s Disease Neuroimaging Initiative (ADNI, https://adni.loni.usc.edu/). Specifically, the discovery dataset was derived from stage 2 (ADNI-2), while the validation dataset was obtained from stage 3 (ADNI-3). The discovery dataset included 133 AD and 157 normal controls (NCs); the validation dataset included 53 AD and 61 NCs. Baseline sMRI, demographic information, and cognitive measures of all subjects were downloaded in de-identified form. Cognitive measures included the Mini-Mental State Examination (MMSE) and four composite cognitive scores for memory function (ADNI-MEM), executive function (ADNI-EF), visuospatial function (ADNI-VS), and language function (ADNI-LAN) (Choi et al., 2020; Crane et al., 2012; Gibbons et al., 2012). In both the discovery and validation datasets, there were no significant differences in age, sex, and number of years of education between AD and NCs (Table 1). However, to account for the potential confounding effects of these variables of no interest, they were used as covariates in the between-group comparison.

**Table 1:** Demographics for all participants

	Features	AD	NCs	Statistics	*p*-value
Discovery dataset (AD = 133, NCs = 157)	Age, mean (SD), and year	74.6(8.1)	73.8(6.4)	1.00	0.32
	Education, mean (SD), and year	15.8(2.7)	16.3(2.5)	−1.77	0.08
	Sex and male/female	77/56	75/82	2.96	0.09
Validation dataset (AD = 53, NCs = 61)	Age, mean (SD), and year	72.7(7.4)	70.4(5.8)	1.85	0.07
	Education, mean (SD), and year	15.3(2.4)	16.1(2.2)	−1.75	0.08
	Sex and male/female	31/22	29/32	1.36	0.24

### T1-weighted image acquisition and preprocessing

T1-weighted (T1w) images were acquired for all subjects using a 3 Tesla scanner. Detailed scan information of T1w images is available on the ADNI website (http://adni.loni.usc.edu/methods/mri-tool/mri-analysis/). The recon-all pre-processing pipeline in FreeSurfer version 7.4.1 software (https://surfer.nmr.mgh.harvard.edu/) was used for T1w image preprocessing, including skull stripping, tissue segmentation, surface reconstruction, metric reconstruction, and spherical normalization parameter estimation (Xue et al., 2023). After that, individual cortical surfaces were reconstructed. A total of four subjects failed to have their T1w images segmented (discovery dataset: 1 patient with AD and 1 NC; validation dataset: 1 patient with AD and 1 NC) after the T1w data pre-processing was completed. Therefore, only the subjects shown in Table 1 were used in this study.

### Construction of MSN gradients

Cortical morphological metrics were extracted using the DK-1533 template (Romero-Garcia, Atienza, Clemmensen, & Cantero, 2012; Yang et al., 2021). The DK-1533 template derives from the Desikan–Killian 68 (DK-68) template (Desikan et al., 2006) and consists of 1533 parcellations with an approximate size of 1 cm^2^ for each parcellation. The DK-1533 template was transformed to each subject’s individual surface space, and five cortical morphological metrics including cortical thickness (CT), cortical volume (CV), cortical area (CA), gaussian curvature (GC), and mean curvature (MC) were extracted for each parcellation. The morphological metrics of each subject were standardized across brain regions (z-score). For each subject, a symmetric matrix of size 1533 × 1533 can be generated by calculating Pearson’s correlation coefficients between the morphological metrics of each pair among the 1533 brain regions. This resulting matrix is referred to as the MSN.

The MSN gradients were generated using the BrainSpace toolbox (Vos de Wael et al., 2020). The main steps are as follows:

1. Referring to previous studies (Hong et al., 2019; Margulies et al., 2016; Vos de Wael et al., 2020; Xia et al., 2022; Xue et al., 2023; Yang et al., 2021), only the top 10% of elements per row in the MSN were retained to compute a cosine similarity matrix that captured similarity in morphological similarity profiles. The similarity matrix underwent an additional transformation into a normalized matrix of angles.
2. We adopted the diffusion map embedding algorithm (Coifman et al., 2005), which is a nonlinear manifold reduction approach, to generate the descending gradient components that explain the MSN variance.
3. The gradient components for each subject were aligned to the template deriving from an average MSN with the usage of the Procrustes rotation (Hong et al., 2019; Xia et al., 2022; Xue et al., 2023).

The first MSN gradient captures the maximum variance in MSN and is along a spectrum from the sensory cortex to the motor cortex (Yang et al., 2021), therefore, we focused only on the first MSN gradient.

### Comparison of the first MSN gradients in AD and NCs

Before making comparisons between groups, we first harmonized for site effects using ComBat (Fortin et al., 2018; Fortin et al., 2017; Johnson, Li, & Rabinovic, 2007). A general linear model (GLM) was conducted to assess the regional MSN gradient differences between AD and NCs, in which age, sex, and educational attainment were covariates. Furthermore, GLM was leveraged to assess the MSN gradient differences between AD and NCs within two other atlases which were referred to as the Yeo functional network atlas (Yeo et al., 2011) and the Von Economo cytoarchitectural class atlas (Economo, Koskinas, & Triarhou, 2008), respectively. Age, sex, and educational attainment were also served as covariates. Yeo atlas consists of seven networks, namely somatomotor network (SOM), visual network (VIS), dorsal attention network (DAN), salience network (SAL), limbic network (LIM), frontoparietal network (FPN), and default mode network (DMN). The Von Economo atlas consists of seven classes, namely primary sensory cortex (Prim sens), secondary sensory cortex (Sec sens), association cortex 1 (Asso1), association cortex 2 (Asso2), limbic regions (Limbic), primary motor cortex (Prim motor), and insula regions (Insula). Kolmogorov–Smirnov test was performed to investigate the differences in the distribution of MSN gradients based on global and cytoarchitectural classes between AD and NCs. *P*-values <0.05 were regarded as statistical significance. *P*-values were adjusted for false discovery rate (FDR) multiple comparisons (Benjamini & Hochberg, 2018).

### Correlation analysis between MSN graph metrics and gradients in AD

We used the Brain Connectivity Toolbox (Rubinov & Sporns, 2010) to calculate two graph metrics, the clustering coefficient, which measures MSN separation, and the average path length, which measures MSN integration (Liao, Vasilakos, & He, 2017; Watts & Strogatz, 1998) (Supplementary Methods). GLM was conducted to assess the AD-NCs differences in the clustering coefficient and the average path length adjusting age, sex, and educational attainment. Graph metrics with statistical significance were further used incorrelation analysis with MSN gradients.

### Correlation analysis between MSN gradients and cognition

The MSN gradients reflect the fundamental properties of cortical organization, and to capture the relationship between the first MSN gradients at the level of Von Economo cytoarchitectural class and cognition in AD, partial correlation analysis was conducted between the first MSN gradients at the level of Von Economo cytoarchitectural class and cognition, with age, sex, and education level as covariates. The gradient within each cytoarchitectural class was computed as the mean of gradients across all brain areas belonging to that class.

### Prediction of cognitive scores

We employed support vector regression, a common supervised machine learning technique, to evaluate the predictive ability of the first MSN gradients for cognition in AD. MSN gradients were the predictor variables, and cognition was the response variable, respectively. We built a nested 5-fold cross-validation framework, and Pearson’s correlation coefficient between observed and predicted cognition was used to assess the predictive performance of the model, and its significance was determined by a permutation test (1 000 times). For comparison with the MSN gradients, we also evaluated the predictive power of the regional MSN (the sum of correlation coefficients for each region) (Supplementary Methods).

### Transcriptomic data and preprocessing

We used the brain transcriptomic datasets from AHBA (Hawrylycz et al., 2012), derived from brain samples of six adult post-mortem donors (Supplementary Table 1). Following the standard workflows (Arnatkeviciute, Fulcher, & Fornito, 2019), we mapped the transcriptomic data to the DK-1533 template using abagen toolbox (Markello et al., 2021), which resulted in a 1533 × 15631 matrix. In brief, the standard workflows include probe re-annotations, filtering, and selection, mapping the transcriptomic data to the DK-1533 template, and data normalization (Supplementary Methods).

### Transcription-neuroimaging association analysis

To investigate the relationship between gene expression profiles and AD-NCs differences in principal MSN gradients (M. Cai et al., 2024; Ma et al., 2023), partial least squares (PLS) regression analysis was conducted where transcriptional profiles were the independent variables and unthresholded t-statistics of the AD-NCs differences in principal MSN gradients were the dependent variables (Supplementary Methods). We focused only on the first PLS component (PLS1), which was a linear combination of transcriptional profiles and was responsible for the most variation in unthresholded t-statistics. Permutation tests (10 000 times) were used to assess the significance of the explanatory variance of PLS1. The significance of the correlation coefficient between PLS1 and unthresholded t-statistics was also established using the permutation test (10 000 times). Bootstrapping (10 000 times) was leveraged to assess the variance of the genes contributing to PLS1, the ratio of the weight for each gene to its standard error (z score) was the corrected weight.

PLS1 genes+ had normalized positive PLS1 weights which indicated high gene expression corresponding to increased alterations in the first MSN gradient in AD compared to NCs, whereas, PLS1 genes- had normalized negative PLS1 weights which indicated low gene expression corresponding to decreased alterations in the first MSN gradient in AD compared to NCs. Gene sets having positive weights (PLS1 genes+) or negative weights (PLS1 genes-) (*p_FDR_* < 0.001) (Shen et al., 2022; Zheng, Xiao, Zhao, Yang, & Guo, 2024a; Zheng, Zhao, et al., 2024b) were retained for enrichment analyses including pathways and Gene Ontology (GO) terms which include biological process (BP), molecular function (MF), and cellular component (CC). Genes enrichment analyses were performed in the ToppGene portal (https://toppgene.cchmc.org/). *P*-values <0.05 were regarded as statistically significant for all enrichment analyses (FDR correction).

### Validation analysis

To validate our main results, we did the following: (i) validating the reproducibility of dominant MSN gradients in a validation dataset; and (ii) adjusting total intracranial volume (TIV) when comparing the first MSN gradient between AD and NCs.

## Results

### Between-group differences in the first MSN gradient

**Figure 1.**
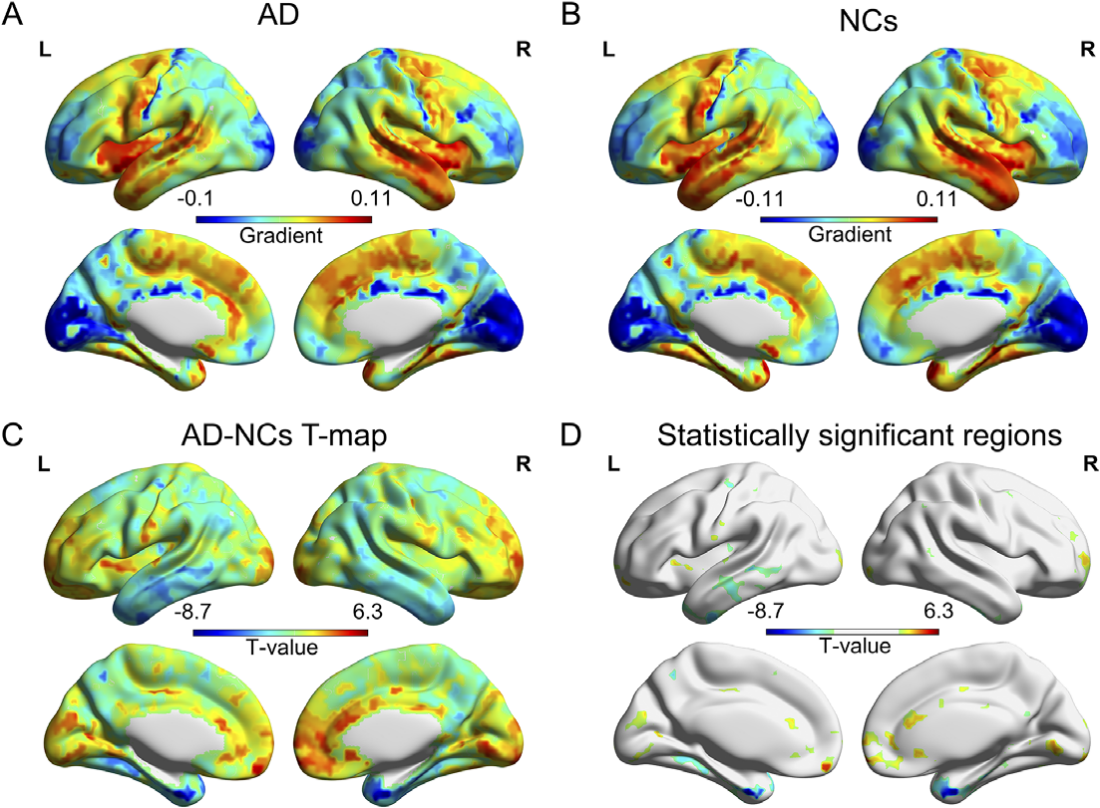
Differences in the first MSN gradient between AD and NCs. A: The first MSN gradient mapping in AD; B: The first MSN gradient mapping in NCs; C: T-statistic map of the differences in the first MSN gradient between AD and NCs; D: brain regions with significant differences in the first MSN gradient between AD and NCs. In C and D, cool colors indicate regions where the gradient is decreased in AD compared to NCs, while warm colors indicate regions where the gradient is increased in AD.

MSN variance explained by the first gradient was not significantly different between AD and NCs (AD, 12.8% ± 0.2%; NCs, 12.8% ± 0.3%, *p* = 0.24, Figure 3A, left). The first MSN gradient exhibited anchoring points at the opposing poles of the motor and sensory cortices, while the association cortex occupied an intermediary position along its continuum (Figure 1A and 1B).

**Figure 2.**
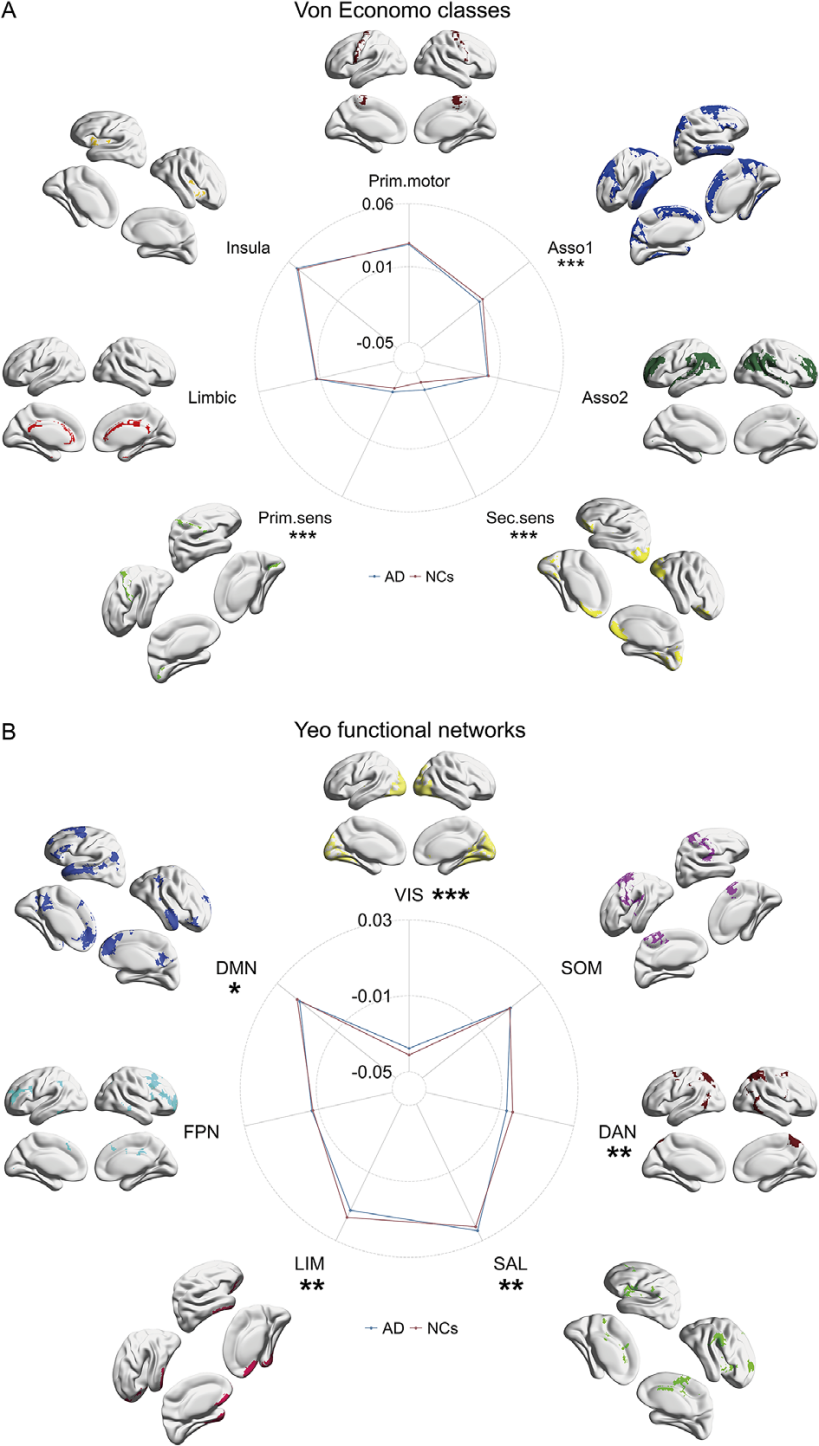
Differences in the first MSN gradient between AD and NCs at the level of Von Economo classes and Yeo functional networks. A: The first MSN gradient differences between AD and NCs at the Von Economo class level; B: The first MSN gradient differences between AD and NCs at the Yeo functional network level. *: *p* < 0.05, **: *p* < 0.01, ***: *p* < 0.001.

Comparisons across different brain regions indicated that in AD patients, the areas showing a significant increase in the first MSN gradient values were predominantly located in the cingulate gyrus, while the regions exhibiting a significant decrease were primarily situated in the temporal lobe (Figure 1D, Supplementary Table 2). Additionally, we utilized two established frameworks for categorizing cortical areas, to broaden the scope of our investigations into the function and cytoarchitecture of cerebral cortex. In patients with AD, at the level of the Von Economo cytoarchitecture class, the first MSN gradient values demonstrate a significant increase in the primary and secondary sensory cortices, while exhibiting a significant decrease in the association cortex 1 (Figure 2A, Supplementary Table 3). In patients with AD, at the level of the Yeo functional networks, the first MSN gradient values demonstrate a significant increase in VIS and SAL, while exhibiting a significant decrease in DAN, LIM, and DMN (Figure 2B, Supplementary Table 4).

The distributions of the first MSN gradient in AD and NCs were significantly different both at the global level (Supplementary Figure 1A, and Supplementary Table 5) and at the level of the Von Economo cytoarchitecture class (Supplementary Figure 1B, and Supplementary Table 5).

### Association between the first MSN gradient and MSN topology in AD

**Figure 3.**
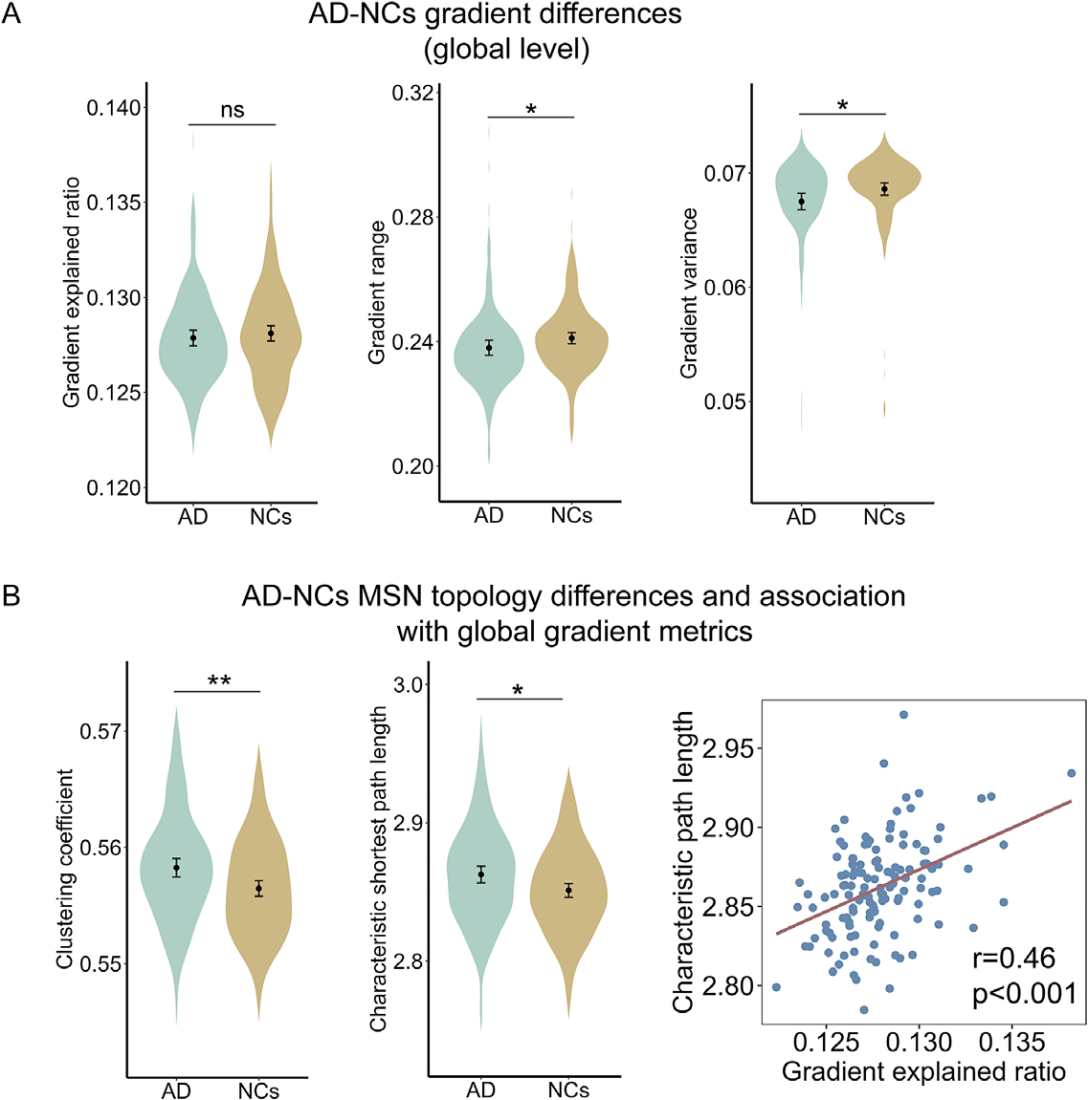
The first MSN gradient and MSN topological properties. A: Differences in global metrics of the first MSN gradient between AD and NCs; B: Differences in MSN topological properties between AD and NCs, and the associations between MSN topological properties and the first MSN gradient. *: *p* < 0.05, **: *p* < 0.01, ns: not significant.

In addition to the first gradient explained ratio, we also compared two other global gradient metrics, the first gradient range, and the first gradient variance. As shown in Figure 3A, middle and right, the first gradient range (AD, 0.238 ± 0.014; NCs, 0.241 ± 0.012, *p* = 0.03) and gradient variance (AD, 0.068 ± 0.004; NCs, 0.069 ± 0.003, *p* = 0.04) of AD were significantly smaller than those of NCs.

The MSN clustering coefficient (AD, 0.558 ± 0.004; NCs, 0.557 ± 0.004, *p* = 0.003) and average path length (AD, 2.862 ± 0.031; NCs, 2.852 ± 0.028, *p* = 0.021) for AD were all significantly larger than NCs (Figure 3B, left and middle). In patients with AD, the average path length and the explained ratio of the first gradient were significantly positively correlated (*r* = 0.46, *p* < 0.001, Figure 3B, right).

### Association between the first MSN gradient and cognition in AD

**Figure 4.**
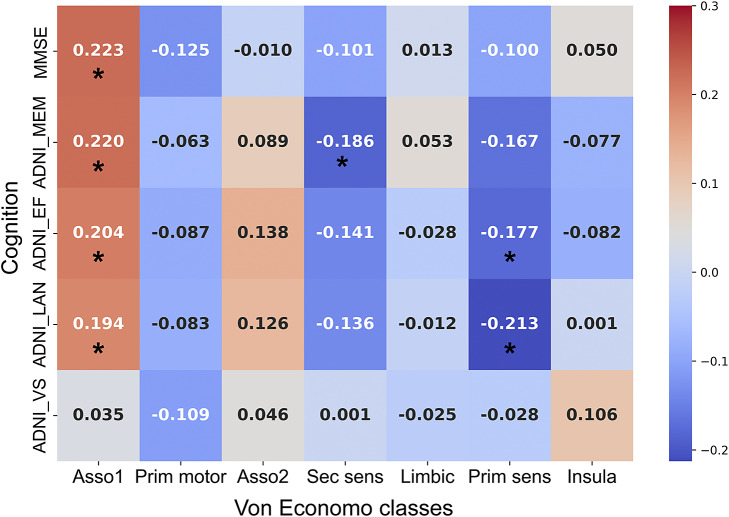
Correlation between the first MSN gradient and cognitive scores in AD. *: *p* < 0.05.

As illustrated in Figure 4, the first MSN gradient of association cortex 1 was significantly positively correlated with executive function (*r* = 0.204, *p* = 0.02), language function (*r* = 0.194, *p* = 0.03), memory function (*r* = 0.220, *p* = 0.01), and MMSE (*r* = 0.223, *p* = 0.01) in AD patients. While the primary sensory cortex was significantly negatively correlated with executive function (*r* = −0.177, *p* = 0.04) and language function (*r* = −0.213, *p* = 0.02), the secondary sensory cortex was significantly negatively correlated with language function (*r* = −0.186, *p* = 0.03).

### The predictive ability of the first MSN gradient on cognition in AD

As shown in Supplementary Table 6, the first MSN gradient was able to significantly predict memory function, executive function, language function, and visual function in AD. Compared with the first MSN gradient, the regional MSN values significantly predicted only executive function, language function, and visual function, and the regional MSN values were less predictive of these cognitive scores than the first MSN gradient was of these cognitive scores.

### Gene expression profiles associated with the first MSN gradient alterations in AD

**Figure 5:**
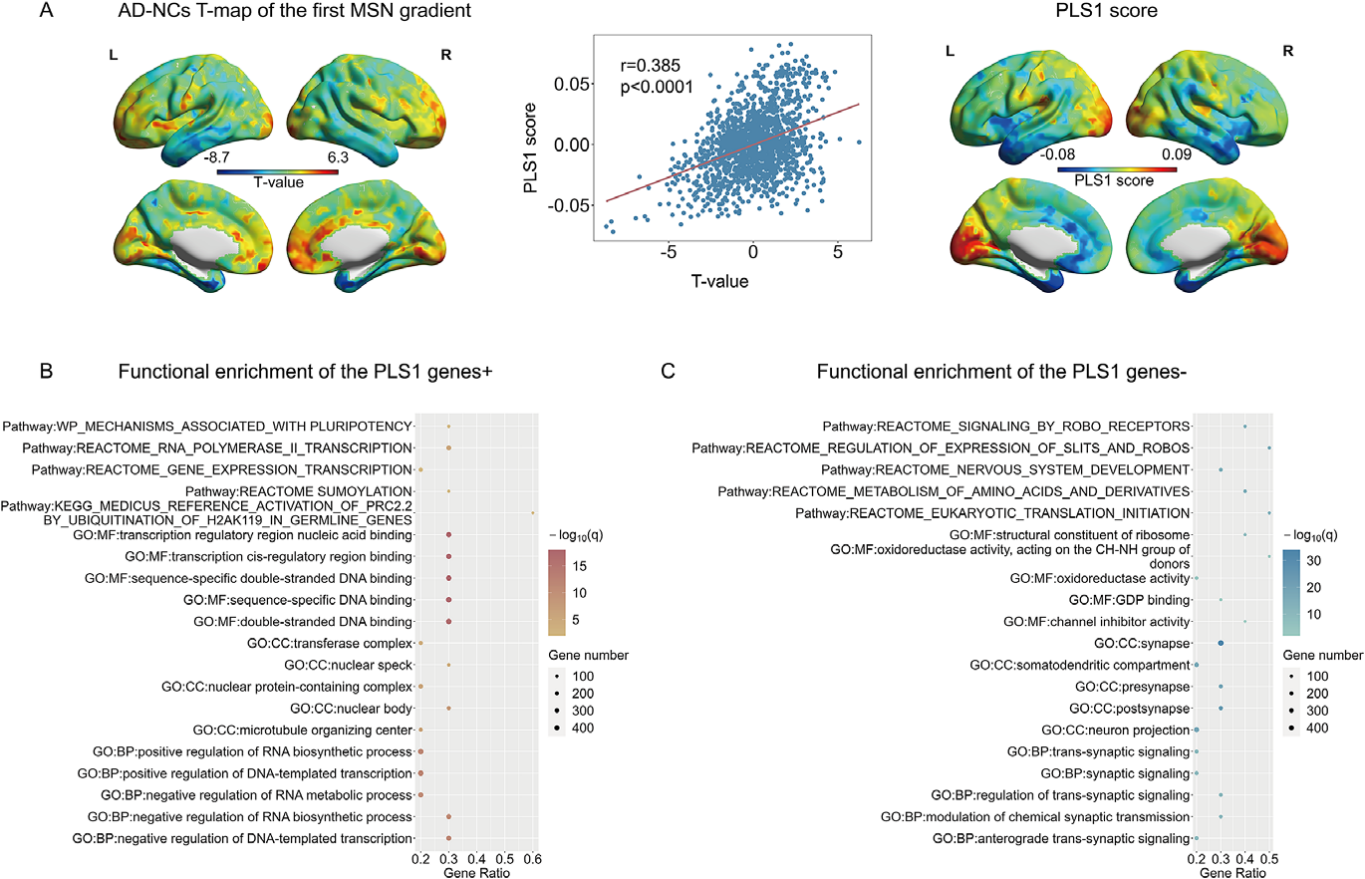
Relationship between regionally altered first MSN gradient in AD and gene expression, alongside enrichment results of PLS1 genes. A: Regional mapping of PLS1 scores, T-map of the first MSN gradient differences between AD and NCs, and correlation between PLS1 scores and the T-map. B: Top enrichment terms for PLS1 genes+. C: Top enrichment terms for PLS1 genes-.

The PLS1 explained 14.8% (*p* < 0.0001) of the variation in AD-associated alterations in the first MSN gradient. Following this, our attention was exclusively channeled towards analyzing the PLS1 in further assessments. The PLS1 displayed distinct transcriptomic patterns, characterized by high expression predominantly in occipital lobe regions and low expression primarily in temporal lobe regions (Figure 5A, right). We also found a significant positive correlation between the PLS1 and between-group t-statistics of the first MSN gradient (*r* = 0.385, *p* < 0.0001) (Figure 5A, middle). Gene set enrichment analysis revealed that PLS1 genes+ were mainly enriched in biological functions and pathways related to gene expression (Figure 5B), whereas PLS1 genes- were mainly enriched in biological processes such as synaptic signaling, molecular functions such as oxidoreductase activity, cellular components such as synapses, and pathways related to metabolism and neural development (Figure 5C).

### Validation results

In the validation set, we obtained the first MSN gradient pattern similar to that of the discovery set, where the motor and sensory cortices were situated at opposite ends, with the association cortex positioned in the middle (Supplementary Figure 2A and Supplementary Figure 2B). Between-group comparisons of the first MSN gradient in the validation set revealed significant brain regions similar to those identified in the discovery set (Supplementary Figure 2D). Specifically, Pearson’s correlation coefficient between the t-statistics obtained from region-based AD-NCs comparisons in the validation set and those from the discovery set was 0.5 (Supplementary Figure 2E). Additionally, when conducting between-group comparisons at the regional level, Pearson’s correlation coefficient between the t-statistics obtained with and without TIV as a covariate exceeded 0.9 (Supplementary Figure 2F).

## Discussion

In this investigation, we have, for the first time, presented the dysfunction of the morphological similarity network hierarchy in AD, and elucidated its correlation with cognitive function and gene expression profiling. We found that the two extremes of the first MSN gradient axis are the sensory cortex and the motor cortex, with the association cortex positioned in the middle. In patients with AD, the abnormal first MSN gradient pattern is primarily characterized by increases in the primary sensory cortex and secondary sensory cortex and decreases in association cortex 1. This pattern is similar to the functional connectome gradient abnormalities observed in AD (Zheng, Zhao, et al., 2024b). In patients with AD, the first MSN gradient in association cortex 1 is positively correlated with functions such as executive functioning, language, and memory, whereas the first gradient in the sensory cortex is negatively correlated with these functions. Furthermore, the first MSN gradient is a significant predictor of cognitive function in AD patients. We also discovered that the genes associated with alterations in the first MSN gradient are predominantly involved in synaptic signaling and metabolic activity. These findings provide new insights into the mechanisms of structural brain network impairment in AD patients, enhancing our understanding of the neurobiological processes responsible for impaired cognition in patients with AD, and offering a new dimensional structural biomarker for AD.

### The first MSN gradient alterations in AD

Compared to group-level structural covariance networks, one of the advantages of MSN is that it integrates multiple morphological features of the cerebral cortex to construct individual-level structural networks (Seidlitz et al., 2018). We applied a neuroimaging phenotype that reflects the continuous spectrum of cortical structural networks – the MSN gradients – to research the differences between AD and NCs. We found that, in both NCs and AD, the sensory cortex and motor cortex are located at the two ends of the first MSN gradient axis, which is consistent with previous studies. They have similar endpoints (Xue et al., 2023; Yang et al., 2021). This differs from the functional network gradient axis, which has unimodal (sensory–motor) regions and transmodal (default mode) regions at its two ends, respectively (Margulies et al., 2016). This indicates that MSN gradients and functional network gradients characterize different hierarchical aspects of the brain from different perspectives. The first MSN gradient pattern is closely related to various fundamental properties of the cortex, such as gene expression (Yang et al., 2021). Compared to healthy controls, AD patients exhibited smaller variance and a narrower range of the first MSN gradient, indicating a smaller disparity in the morphological connectivity patterns between the sensory and motor cortices in AD patients. The regions exhibiting increased first MSN gradient in patients with AD are primarily localized within the frontal and cingulate gyrus, whereas decreased regions are predominantly situated within the temporal lobe. Research has indicated that the medial temporal lobe is affected even before cognitive decline becomes apparent in AD (Burggren & Brown, 2014). Our findings further support the crucial role of the temporal lobe in the pathogenesis of AD from a network hierarchy perspective.

On the Yeo functional network, this is reflected by an increase in the first MSN gradient in the VIS and a decrease in the first MSN gradient in networks such as DMN and LIM. In the class of Von Economo, there is a notable increase in the first MSN gradient primarily in the primary and secondary sensory cortices, while there is a decrease in the first MSN gradient in the association cortex 1. The VIS receives external signals and filters relevant information before transmitting the signals to higher-order systems to induce appropriate responses. Functional alterations in the VIS occur even before the onset of cognitive deficits in AD and worsen as the disease progresses (Devanand et al., 2008; Lin et al., 2011; Verghese, Wang, Lipton, Holtzer, & Xue, 2007). Disruption of cortical connectivity in AD leads to insufficient bottom-up audiovisual integration (Festa, Katz, Ott, Tremont, & Heindel, 2017). Functional impairments in the DMN are also associated with the pathophysiology of memory deficits and various cognitive impairments in AD (Ibrahim et al., 2021; Mandal, Banerjee, Tripathi, & Sharma, 2018). The pattern of changes in the first MSN gradient observed in this study parallels the alterations in the functional network gradients in AD (Zheng, Zhao, et al., 2024b). This finding supports previous observations of deficits in early sensory and visual processing and highlights the importance of the DMN in AD, from the perspective of continuity of cortical spatial organization.

### The relationship between the first MSN gradient and cognition

We also found that association cortex 1 is positively correlated with functions such as memory, language, and executive functions, whereas the primary sensory cortex is negatively correlated with these functions. Furthermore, the first MSN gradient can significantly predict memory, language, executive, and visual functions in AD patients. This suggests that alterations in the first MSN gradient may be an endophenotype of AD pathology. The study also discovered that both integration and segregation of MSN are increased in AD patients, and there is a notable association between these topological changes of MSN and the first MSN gradient.

### Transcription-neuroimaging association analysis

Our transcription-neuroimaging association analysis revealed a connection between AD-related changes in the first MSN gradient and gene expression enriched in synaptic signaling and metabolic activity. This suggests that synaptic signaling and metabolic activity may be potential mechanisms underlying the alterations in the principal MSN gradient in AD. Synaptic dysfunction plays a crucial role in the pathology of AD (Selkoe, 2002). Trans-synaptic signaling is one of the most fundamental biological processes, involving a series of critical molecular functions, including synapse formation and the regulation of synaptic plasticity (de Wit & Ghosh, 2016; Fossati et al., 2019). Disruption of synaptic signaling across numerous important pathways affects synapse formation and stability, which is pivotal in the pathology of AD (Selkoe, 2002). Metabolic abnormalities are key characteristics of the neuronal cellular milieu in AD, including impaired energy metabolism, disrupted lipid metabolism, and decreased overall metabolic capacity. These abnormalities have the potential to cause instability in neural network activity, impair neuroplasticity, and disrupt the hierarchical organization of the brain, thereby undermining the harmonious functioning of the brain (Xu, Liu, Qin, & Wang, 2023). Genes associated with changes in functional network gradients are enriched in ion transmembrane transporter activity (Zheng, Zhao, et al., 2024b), while genes related to changes in the first MSN gradients are not enriched in this biological function. We cautiously infer that the reason for this difference may be that functional network gradients and the first MSN gradients explore the hierarchical nature of the brain from different aspects, respectively. Compared to NCs, the alterations in functional gradients and the first MSN gradients at the region level are distinct in AD. Moreover, neuroimaging-transcriptome association analysis is based on PLS regression between T-values of region-level differences in gradients and gene expression values, which may lead to different results in gene enrichment. Given the current challenges in the measurement of the expression of regional genes in the living brain, the findings of this study offer valuable clues for understanding the relationship between microscopic biological events and the macrostructural changes observed in AD.

### Limitations

The current study has several limitations. First, although the data analyzed in this article comes from public databases, the participants’ race or ethnicity information is unavailable, and we are unsure whether there are any racial or ethnic differences, which is a limitation of this study. Second, indicators of AD-related pathology, such as beta-amyloid and tau proteins, were not included. Future research should incorporate these indicators to assess how they affect the MSN gradient associated with AD. Third, gene expression datasets are from donors without a diagnosis of AD. Additional transcriptomic data from patients with AD was essential to link MSN gradients to transcriptomic profiles. To elucidate the genetics underlying AD-associated gradient changes, genome-wide association studies are needed in future studies to examine the synergistic roles of related genes in the pathogenesis of AD, given its complex polygenic nature. Finally, besides genetic factors, environmental influences are also crucial. Subsequent investigations ought to prioritize exploring exposome-neuroimaging relationships to advance our comprehension of the etiological contributors influencing MSN gradients in AD (F. Liu et al., 2023).

In conclusion, the findings of our study support the hypotheses that changes in the first MSN gradient are present in patients with AD, and these changes are correlated with cognitive scores and transcriptomic profiles. Additionally, our study showed that the genes involved in AD showed an enrichment in pathways that were relevant to neurobiology. Collectively, our findings shed new light on the altered structural coordination in AD and had the potential to provide a novel endophenotype for further exploration of complicated mechanisms underlying AD.

## Supporting information

Zheng et al. supplementary material Zheng et al. supplementary material

## Data Availability

The sMRI and cognition data were obtained from ADNI (https://adni.loni.usc.edu/), and gene expression data were obtained from AHBA (http://human.brain-map.org/static/download).

## Supplementary material

The supplementary material for this article can be found at http://doi.org/10.1017/S0033291725000091.
