# Supplementary material for: Dysfunction in the hierarchy of morphometric similarity network in Alzheimer’s disease and its correlation with cognitive performance and gene expression profiles
Source: Psychol Med. 2025 Feb 12;55:e42. doi: 10.1017/S0033291725000091 (PMC12055026; doi:10.1017/S0033291725000091)
Supplement: Zheng et al. supplementary material [file S0033291725000091sup001.docx]

**Dysfunction in the hierarchy of morphometric similarity network in Alzheimer's disease and its correlation with cognitive performance and gene expression profiles**

Chuchu Zheng, Ph.D.^1,2,3^, Wei Zhao, Ph.D.^2,3^, Zeyu Yang, B.S^2,3^, Shuixia Guo, Ph.D. ^2,3^*, the Alzheimer's Disease Neuroimaging Initiative^^[[1]](#footnote-1)^†^

**Author affiliations:**

1 School of Public Health, Shanxi Medical University, Taiyuan, Shanxi, 030001, People’s Republic of China.

2 MOE-LCSM, School of Mathematics and Statistics, Hunan Normal University, Changsha, Hunan, 410006, People’s Republic of China.

3 Key Laboratory of Applied Statistics and Data Science, Hunan Normal University, College of Hunan Province, Changsha, Hunan, 410006, People’s Republic of China.

*Correspondence to:

Shuixia Guo, Ph.D.

MOE-LCSM, School of Mathematics and Statistics, Hunan Normal University, Changsha, Hunan, 410006, People’s Republic of China.

E-mail: [guoshuixia75@163.com](mailto:guoshuixia75@163.com)

Contents

[Supplementary Methods 3](#_Toc184249537)

[Calculation of MSN graph metrics 3](#_Toc184249538)

[Prediction of cognitive scores 4](#_Toc184249539)

[Gene expression data preprocessing 5](#_Toc184249540)

[Partial least squares regression analysis 6](#_Toc184249541)

[Supplementary Tables 7](#_Toc184249542)

[Supplementary Table 1. Information on all donors in the Allen Human Brain Atlas database 7](#_Toc184249543)

[Supplementary Table 2. Regional differences in the first MSN gradient between AD and NCs 7](#_Toc184249544)

[Supplementary Table 3. The differences in the first MSN gradient in each functional network 12](#_Toc184249545)

[Supplementary Table 4. The differences in the first MSN gradient in each cytoarchitectural class 12](#_Toc184249546)

[Supplementary Table 5. The differences in the first MSN gradient distribution 12](#_Toc184249547)

[Supplementary Table 6. Prediction of cognitive scores in AD 13](#_Toc184249548)

[Supplementary Figures 13](#_Toc184249549)

[Supplementary Figure 1 Distribution of the first MSN gradient in AD and NCs 13](#_Toc184249550)

[Supplementary Figure 2 Results of the robustness analysis 14](#_Toc184249551)

[References 15](#_Toc184249552)

# Supplementary Methods

## Calculation of MSN graph metrics

In this study, we used the Brain Connectivity Toolbox(Rubinov & Sporns, 2010) to produce two important MSN graph metrics, The MSN sparsity threshold value used to compute the graph metrics was the same as that used to compute the gradients:

(1) Clustering coefficient (C): This metric quantifies brain network segregation. In a simple graph, let the neighbor set of node *i* be$N(i)$, notation$\left|N(i)\right|=k_{i}$. Then the clustering coefficient of node *i* is defined as the ratio of the actual number of edges $E_{i}$ that exist between these $k_{i}$ nodes to the total number of edges $\frac{k_{i}(k_{i}-1)}{2}$ that can exist:

$$C_{i}=\frac{2E_{i}}{k_{i}(k_{i}-1)}$$

The clustering coefficient of the whole network will then be the average of the clustering coefficients of all the nodes in the network:

$$C=\frac{1}{N}\sum_{i=1}^{N} C_{i}$$

Where *N* is the total number of nodes in the network, the larger *C* is, the higher the degree of segregation of the network.

(2) Characteristic path length (L): Characteristic path length is also known as average path length which quantifies brain network integration. The path with the least number of edges between two nodes in the network is called the shortest path, and the length of the shortest path is then the distance between the two nodes. Let the shortest path between node *i* and node *j* be$d_{ij}$. Then the characteristic path length of the network is the average of the distances between all pairs of nodes in the network:

$$L=\frac{1}{\frac{1}{2}N(N-1)}\sum d_{ij}$$

Where *N* is the total number of nodes in the network and the smaller *L* is, the higher the degree of integration of the network.

## Prediction of cognitive scores

We used a supervised machine learning method, linear kernel support vector regression, to assess the predictive ability of the first MSN gradients on cognitive scores in patients with AD. The first MSN gradients are the predictor variable and the cognitive scores (MMSE, ADNI_MEM, ADNI _EF, ADNI _LAN, ADNI _VS) are the response variable. In this study, a nested 5-fold cross-validation framework was established where external 5-fold cross-validation was used to estimate the predictive performance of the model and internal 5-fold cross-validation was used to optimize the model hyper-parameters. In the external 5-fold cross-validation, all the data were randomly divided into five subsets, four of which served as the training set and one of the remaining subsets served as the validation set, so that it was looped five times sequentially without repetition so that each subset was performed as a test set. For each cycle of external 5-fold cross-validation, an internal 5-fold cross-validation is performed to optimize the parameters of the support vector regression by grid search. In this study, the penalty coefficients were optimized with values ranging from 2^-5^-2^10^.To avoid bias caused by features with a wide range of values, each feature in the training dataset was normalized (z-score) and the estimated parameters were applied to the test dataset(Xia et al., 2022). Prediction accuracy was evaluated as the Pearson’s correlation coefficient between predicted and observed cognitive scores for all AD patients. Statistical significance of the predictive accuracy evaluation metrics was assessed by permutation test (1000 times). This predictive analysis was mainly performed using libsvm (www.csie.ntu.edu.tw/~cjlin/libsvm/) and Cui's code (https://github.com/ZaixuCui/Pattern_Regression_Matlab). In addition, to compare with the predictive ability of the first MSN gradients, this study also evaluated the predictive ability of the regional MSN value, which for each brain region is the sum of Pearson's correlation coefficients between that brain region and all the others, for example, the regional MSN value of node *i* can be expressed as:

$$MSN_{i}=\sum_{j\in N} r_{ij}$$

where *N* is the number of nodes in the network and $r_{ij}$ is the Pearson's correlation coefficient between the morphological features of node i and any node *j* in the network.

## Gene expression data preprocessing

The whole-brain gene expression data were derived from Allen Human Brain Atlas (AHBA) (https://portal.brain-map.org/)(Hawrylycz et al., 2012). The brain tissue samples in the AHBA were obtained from six adult donors who had no known history of neuropsychiatric or neurological conditions. Details of the six donors in AHBA were presented in Supplementary Table 1. Based on the recommended processing pipeline (Arnatkeviciute, Fulcher, & Fornito, 2019), we preprocessed the gene expression data of the human brain tissue samples deriving AHBA by applying abagen toolbox (Markello et al., 2021). The preprocessing procedure can be summarized as follows:

(1) Probe-to-gene re-annotations: with each update of the sequencing database, the annotation tables provided in AHBA for mapping probes to genes become outdated, and accurate probe-to-gene annotations are essential for obtaining biologically meaningful discoveries. Therefore, it is necessary to reassign probes to genes using the latest available sequencing information;

(ii) Data filtering: due to nonspecific hybridization, microarray experiments are prone to background noise, so appropriate controls must be used to distinguish between expression signal and noise. We filtered annotated probes according to their intensity that did not exceed background noise in 50% of all tissue samples;

(iii) Probe selection: the probe with the highest differential stability was selected to represent the gene;

(iv) Assigning samples to brain regions: assign samples to brain regions in a single parcellation (DK-1533 atlas in our study) defined in Montreal Neurological Institute (MNI) space via the nearest area found within a 2mm Euclidean distance. The empty regions in the parcellation will be assigned the expression values of the tissue sample falling closest to the centroid of that region;

(v) Standardizing data: including sample normalization and gene normalization. Finally, 15631 genes from 1533 cortical regions were available for downstream analysis.

## Partial least squares regression analysis

Currently, measuring regional gene expression in the brain in vivo is extremely difficult. The gene expression data used in our study were derived from donors not diagnosed with AD in AHBA. Thus, we used a partial least squares (PLS) regression analysis to explore the association between the transcriptional profiles and AD-NCs differences in the first MSN gradient map. PLS regressions can define several components, and the first component (PLS1) is a linear combination of the predictor variables (gene expression data) that can explain most of the variance in the response variables(t-statistics). PLS analysis has also been widely used in transcription-neuroimaging association analysis(Shen et al., 2022; Xia et al., 2022; Zheng, Xiao, Zhao, Yang, & Guo, 2024; Zheng, Zhao, Yang, & Guo, 2024).

The general model for partial least squares regression is as follows:

$$X=RP^{T}+E$$

$$Y=UQ^{T}+F$$

Where *X* is an *n × m* prediction matrix, *Y* is an *n × p* response matrix, *R* is an *n × l* score matrix (projections on *P*), and *U* is an *n × l* score matrix (projections on *Q*). *P* is the *m × l* orthogonal load matrix, and *Q* is the *p × l* orthogonal load matrix. *E* and *F* are the error matrices, respectively.

In our study, *X* is a gene expression matrix (that is 1533 regions×15631 genes for cortical regions). *Y* is a vector of unthresholded t-statistics obtained from between-group comparisons (AD vs. NCs) of the first MSN gradients. The reason for using unthresholded t-statistics as *Y* is that we aim to explore the genetic mechanism underlying the first MSN gradient differences between AD and NCs. The t-statistics represent the between-group (AD vs. NCs) differences in the first MSN gradients.

Our goal is to find a pair of *X* and *Y* decompositions such that the *R* and *U* covariance is maximum. In other words, we find the direction of *P* and *Q* such that *X* and *Y* are most strongly correlated in that direction. Each vector in *P* and *Q* is such a direction (or principal component). The number of principal components is *l*.

# Supplementary Tables

## Supplementary Table 1. Information on all donors in the Allen Human Brain Atlas database

| **Donor ID** | **Sex** | **Age** | **Postmortem interval** | **Handedness** | **Hemispheres** | **Samples** | **Medical information** |
| --- | --- | --- | --- | --- | --- | --- | --- |
| H0351.2001 | Male | 24 | 23 h | Left | Left + Right | 946 | History of asthma |
| H0351.2002 | Male | 39 | 10 h | Left | Left + Right | 893 | None known |
| H0351.1009 | Male | 57 | 25.5 h | Cross-dominant | Left | 363 | History of atherosclerotic cardiovascular disease |
| H0351.1012 | Male | 31 | 17.5 h | Right | Left | 529 | Sudden cardiac arrest. Benign spindle cell proliferation and dystrophic calcification in the temporal horn of the lateral ventricle, ~5 mm, possibly an old choroid plexus infarct or degenerated xanthogranuloma |
| H0351.1015 | Female | 49 | 30 h | Right | Left | 470 | Splenectomy, hypothyroidism treated with Levothroid |
| H0351.1016 | Male | 55 | 18 h | Right | Left | 501 | Coronary artery atherosclerosis, prescriptions for clotting, and high  cholesterol |

## Supplementary Table 2. Regional differences in the first MSN gradient between AD and NCs

| Region | AD | NCs | t-statistic | *p_FDR_* |
| --- | --- | --- | --- | --- |
| lh_caudalanteriorcingulate_part4_area | 0.010(0.069) | -0.022(0.066) | 3.95 | 3.00E-03 |
| lh_cuneus_part4_area | -0.093(0.026) | -0.101(0.024) | 3.14 | 0.024 |
| lh_cuneus_part5_area | -0.094(0.027) | -0.102(0.023) | 3.15 | 0.024 |
| lh_cuneus_part6_area | -0.089(0.025) | -0.097(0.024) | 3.31 | 0.016 |
| lh_cuneus_part11_area | -0.086(0.023) | -0.096(0.022) | 4.11 | 2.00E-03 |
| lh_cuneus_part12_area | -0.097(0.016) | -0.104(0.018) | 3.14 | 0.024 |
| lh_entorhinal_part2_area | -0.010(0.063) | 0.045(0.050) | -8.69 | 5.11E-13 |
| lh_entorhinal_part3_area | -0.069(0.031) | -0.042(0.041) | -6.95 | 9.65E-09 |
| lh_entorhinal_part4_area | -0.003(0.063) | 0.052(0.052) | -8.39 | 1.79E-12 |
| lh_entorhinal_part5_area | 0.030(0.071) | 0.056(0.053) | -3.69 | 0.006 |
| lh_fusiform_part3_area | 0.019(0.063) | 0.04(0.047) | -3.26 | 0.019 |
| lh_fusiform_part11_area | -0.001(0.068) | 0.031(0.057) | -4.48 | 1.00E-03 |
| lh_fusiform_part18_area | -0.001(0.071) | 0.03(0.057) | -4.07 | 2.00E-03 |
| lh_fusiform_part20_area | -0.025(0.058) | 0.004(0.057) | -4.43 | 1.00E-03 |
| lh_fusiform_part27_area | 0.033(0.065) | 0.054(0.053) | -3.13 | 0.024 |
| lh_inferiorparietal_part2_area | -0.034(0.05) | -0.018(0.053) | -2.92 | 0.038 |
| lh_inferiorparietal_part26_area | -0.001(0.07) | 0.025(0.056) | -3.58 | 0.008 |
| lh_inferiortemporal_part5_area | 0.045(0.064) | 0.066(0.055) | -3.11 | 0.025 |
| lh_inferiortemporal_part6_area | 0.034(0.048) | 0.054(0.041) | -4.27 | 1.00E-03 |
| lh_inferiortemporal_part8_area | 0.036(0.062) | 0.055(0.050) | -3.04 | 0.029 |
| lh_inferiortemporal_part9_area | 0.023(0.064) | 0.054(0.048) | -4.94 | 1.49E-04 |
| lh_inferiortemporal_part10_area | 0.015(0.064) | 0.035(0.056) | -2.85 | 0.044 |
| lh_inferiortemporal_part16_area | -0.017(0.06) | 0.008(0.061) | -3.85 | 4.00E-03 |
| lh_inferiortemporal_part19_area | 0.035(0.061) | 0.015(0.066) | 2.99 | 0.033 |
| lh_inferiortemporal_part23_area | -0.009(0.06) | 0.011(0.053) | -3.34 | 0.015 |
| lh_inferiortemporal_part25_area | 0.018(0.058) | 0.036(0.051) | -3.15 | 0.024 |
| lh_inferiortemporal_part27_area | 0.016(0.060) | 0.036(0.058) | -2.88 | 0.041 |
| lh_isthmuscingulate_part1_area | -0.002(0.079) | 0.023(0.070) | -3.21 | 0.021 |
| lh_lateraloccipital_part21_area | -0.067(0.045) | -0.083(0.034) | 3.3 | 0.017 |
| lh_lateraloccipital_part27_area | -0.076(0.040) | -0.089(0.027) | 3.72 | 0.005 |
| lh_lateraloccipital_part32_area | -0.090(0.029) | -0.099(0.020) | 3.24 | 0.019 |
| lh_lateraloccipital_part38_area | -0.079(0.037) | -0.092(0.032) | 3.08 | 0.027 |
| lh_lingual_part18_area | -0.096(0.017) | -0.102(0.017) | 2.94 | 0.036 |
| lh_lingual_part21_area | -0.082(0.038) | -0.099(0.022) | 4.83 | 2.16E-04 |
| lh_lingual_part22_area | -0.078(0.033) | -0.091(0.028) | 3.72 | 0.006 |
| lh_lingual_part26_area | -0.082(0.035) | -0.095(0.027) | 3.15 | 0.024 |
| lh_lingual_part27_area | -0.094(0.025) | -0.103(0.017) | 3.28 | 0.017 |
| lh_lingual_part28_area | -0.089(0.031) | -0.1(0.025) | 3.00 | 0.032 |
| lh_medialorbitofrontal_part10_area | -0.004(0.059) | -0.037(0.058) | 5.11 | 8.05E-05 |
| lh_medialorbitofrontal_part12_area | -0.016(0.061) | -0.036(0.053) | 3.04 | 0.029 |
| lh_middletemporal_part1_area | -0.001(0.060) | 0.021(0.056) | -3.18 | 0.022 |
| lh_middletemporal_part4_area | 0.051(0.058) | 0.076(0.039) | -4.14 | 2.00E-03 |
| lh_middletemporal_part8_area | 0.035(0.060) | 0.055(0.044) | -2.96 | 0.035 |
| lh_middletemporal_part12_area | 0.008(0.057) | 0.032(0.051) | -4.06 | 2.00E-03 |
| lh_middletemporal_part14_area | 0.020(0.061) | 0.042(0.053) | -3.79 | 4.00E-03 |
| lh_middletemporal_part15_area | 0.036(0.065) | 0.063(0.05) | -4.78 | 2.28E-04 |
| lh_middletemporal_part16_area | -1.77E-4(0.052) | 0.013(0.046) | -2.82 | 0.047 |
| lh_middletemporal_part18_area | 0.003(0.059) | 0.021(0.048) | -3.37 | 0.014 |
| lh_middletemporal_part19_area | 0.030(0.054) | 0.049(0.045) | -3.51 | 0.010 |
| lh_middletemporal_part20_area | 0.029(0.057) | 0.058(0.044) | -4.63 | 3.78E-04 |
| lh_middletemporal_part21_area | 0.008(0.052) | 0.025(0.050) | -2.84 | 0.045 |
| lh_middletemporal_part22_area | 0.052(0.044) | 0.067(0.039) | -3.52 | 0.010 |
| lh_parahippocampal_part1_area | 0.079(0.052) | 0.094(0.033) | -2.85 | 0.044 |
| lh_parahippocampal_part2_area | 0.068(0.056) | 0.092(0.035) | -4.59 | 4.21E-04 |
| lh_parahippocampal_part3_area | 0.059(0.057) | 0.078(0.044) | -3.30 | 0.017 |
| lh_parahippocampal_part5_area | 0.078(0.051) | 0.096(0.026) | -3.74 | 0.005 |
| lh_parahippocampal_part7_area | 0.068(0.054) | 0.083(0.038) | -2.95 | 0.035 |
| lh_pericalcarine_part1_area | -0.085(0.022) | -0.096(0.021) | 4.09 | 2.00E-03 |
| lh_pericalcarine_part3_area | -0.079(0.022) | -0.088(0.021) | 4.18 | 2.00E-03 |
| lh_pericalcarine_part4_area | -0.097(0.017) | -0.104(0.020) | 2.90 | 0.039 |
| lh_postcentral_part14_area | -0.010(0.060) | 0.011(0.060) | -3.05 | 0.029 |
| lh_postcentral_part24_area | -3.52E-4(0.060) | -0.019(0.062) | 3.53 | 0.009 |
| lh_postcentral_part39_area | -0.002(0.063) | -0.022(0.064) | 3.53 | 0.009 |
| lh_posteriorcingulate_part5_area | 0.034(0.061) | 0.004(0.059) | 4.05 | 2.00E-03 |
| lh_posteriorcingulate_part12_area | 0.026(0.068) | 0.001(0.063) | 3.54 | 0.009 |
| lh_precentral_part21_area | -0.040(0.046) | -0.054(0.039) | 3.23 | 0.020 |
| lh_rostralanteriorcingulate_part1_area | 0.023(0.066) | -0.003(0.065) | 3.21 | 0.021 |
| lh_rostralanteriorcingulate_part5_area | 0.015(0.060) | -0.007(0.061) | 3.19 | 0.022 |
| lh_rostralmiddlefrontal_part17_area | 0.018(0.060) | -0.009(0.060) | 4.08 | 2.00E-03 |
| lh_rostralmiddlefrontal_part32_area | -0.028(0.059) | -0.049(0.049) | 3.43 | 0.012 |
| lh_rostralmiddlefrontal_part34_area | -0.033(0.064) | -0.055(0.047) | 3.10 | 0.026 |
| lh_rostralmiddlefrontal_part38_area | -0.056(0.046) | -0.070(0.036) | 2.99 | 0.032 |
| lh_superiorfrontal_part2_area | -0.031(0.057) | -0.050(0.051) | 3.37 | 0.014 |
| lh_superiorfrontal_part10_area | -0.014(0.059) | -0.036(0.059) | 3.43 | 0.013 |
| lh_superiorfrontal_part53_area | 0.016(0.069) | 0.039(0.063) | -2.95 | 0.036 |
| lh_superiorparietal_part19_area | -0.027(0.054) | -0.041(0.049) | 2.83 | 0.046 |
| lh_superiortemporal_part1_area | 0.036(0.054) | 0.056(0.045) | -3.68 | 0.006 |
| lh_superiortemporal_part7_area | 0.008(0.058) | 0.028(0.049) | -3.95 | 3.00E-03 |
| lh_superiortemporal_part24_area | 0.060(0.041) | 0.082(0.032) | -4.78 | 2.28E-04 |
| lh_superiortemporal_part25_area | 0.036(0.050) | 0.058(0.043) | -4.33 | 1.00E-03 |
| lh_superiortemporal_part26_area | 0.062(0.041) | 0.075(0.030) | -2.98 | 0.033 |
| lh_superiortemporal_part30_area | 0.053(0.044) | 0.074(0.034) | -4.63 | 3.78E-04 |
| lh_superiortemporal_part31_area | -0.014(0.039) | 0.007(0.037) | -4.87 | 1.88E-04 |
| lh_superiortemporal_part34_area | 0.050(0.045) | 0.075(0.030) | -5.52 | 1.19E-05 |
| lh_frontalpole_part2_area | -0.016(0.059) | -0.036(0.054) | 2.91 | 0.038 |
| lh_temporalpole_part3_area | 0.055(0.041) | 0.079(0.024) | -6.41 | 1.54E-07 |
| lh_temporalpole_part4_area | 0.041(0.045) | 0.060(0.034) | -4.43 | 1.00E-03 |
| lh_insula_part6_area | 0.042(0.057) | 0.064(0.039) | -4.05 | 2.00E-03 |
| lh_insula_part20_area | 0.069(0.049) | 0.044(0.061) | 4.07 | 2.00E-03 |
| rh_caudalanteriorcingulate_part1_area | -0.078(0.034) | -0.089(0.024) | 3.19 | 0.022 |
| rh_caudalanteriorcingulate_part3_area | 2.17E-4(0.063) | -0.029(0.062) | 4.07 | 2.00E-03 |
| rh_caudalanteriorcingulate_part4_area | 0.071(0.048) | 0.053(0.060) | 2.93 | 0.037 |
| rh_caudalanteriorcingulate_part7_area | -0.003(0.066) | -0.030(0.062) | 3.96 | 3.00E-03 |
| rh_caudalmiddlefrontal_part19_area | -2.86E-4(0.061) | 0.018(0.054) | -2.99 | 0.032 |
| rh_cuneus_part10_area | -0.052(0.048) | -0.067(0.044) | 3.02 | 0.031 |
| rh_entorhinal_part1_area | -0.017(0.057) | 0.022(0.052) | -6.25 | 2.90E-07 |
| rh_entorhinal_part2_area | -0.019(0.055) | 0.031(0.051) | -8.29 | 2.33E-12 |
| rh_entorhinal_part3_area | 0.001(0.064) | 0.036(0.060) | -4.78 | 2.28E-04 |
| rh_entorhinal_part4_area | -0.031(0.05) | -0.006(0.054) | -4.11 | 2.00E-03 |
| rh_fusiform_part2_area | 0.015(0.066) | 0.039(0.058) | -3.33 | 0.015 |
| rh_fusiform_part5_area | 0.015(0.066) | 0.045(0.047) | -4.18 | 2.00E-03 |
| rh_fusiform_part14_area | 0.039(0.057) | 0.019(0.061) | 2.99 | 0.032 |
| rh_fusiform_part16_area | 0.005(0.061) | 0.028(0.054) | -3.22 | 0.02 |
| rh_inferiorparietal_part2_area | 0.013(0.051) | 0.026(0.048) | -2.90 | 0.039 |
| rh_inferiorparietal_part4_area | -0.015(0.052) | 0.003(0.049) | -3.14 | 0.024 |
| rh_inferiorparietal_part7_area | -0.044(0.050) | -0.030(0.051) | -2.98 | 0.033 |
| rh_inferiorparietal_part20_area | -0.027(0.061) | -0.008(0.058) | -3.03 | 0.03 |
| rh_inferiorparietal_part28_area | -0.007(0.066) | 0.017(0.058) | -3.57 | 0.008 |
| rh_inferiorparietal_part33_area | -0.025(0.054) | -0.006(0.055) | -3.34 | 0.015 |
| rh_inferiortemporal_part18_area | 0.044(0.050) | 0.062(0.045) | -3.10 | 0.026 |
| rh_inferiortemporal_part21_area | 0.034(0.070) | 0.065(0.054) | -4.20 | 2.00E-03 |
| rh_inferiortemporal_part22_area | -0.003(0.062) | 0.023(0.062) | -3.71 | 0.006 |
| rh_isthmuscingulate_part1_area | -0.014(0.067) | 0.008(0.060) | -3.42 | 0.013 |
| rh_lateraloccipital_part4_area | -0.086(0.030) | -0.095(0.027) | 3.37 | 0.014 |
| rh_lateraloccipital_part17_area | -0.070(0.042) | -0.089(0.032) | 4.33 | 1.00E-03 |
| rh_lateraloccipital_part27_area | -0.029(0.065) | -0.052(0.051) | 3.17 | 0.023 |
| rh_lateraloccipital_part33_area | -0.062(0.047) | -0.080(0.034) | 4.14 | 2.00E-03 |
| rh_lateraloccipital_part40_area | -0.046(0.064) | -0.069(0.049) | 3.03 | 0.03 |
| rh_lateraloccipital_part42_area | -0.052(0.050) | -0.069(0.047) | 3.04 | 0.03 |
| rh_lingual_part13_area | -0.062(0.046) | -0.082(0.042) | 4.27 | 1.00E-03 |
| rh_lingual_part19_area | -0.078(0.044) | -0.095(0.033) | 3.7 | 0.006 |
| rh_lingual_part22_area | -0.080(0.035) | -0.095(0.030) | 3.46 | 0.011 |
| rh_lingual_part26_area | -0.098(0.019) | -0.107(0.016) | 4.45 | 1.00E-03 |
| rh_lingual_part27_area | -0.070(0.050) | -0.086(0.040) | 2.83 | 0.046 |
| rh_lingual_part28_area | -0.094(0.021) | -0.102(0.018) | 3.5 | 0.01 |
| rh_medialorbitofrontal_part6_area | -0.010(0.064) | -0.039(0.060) | 3.97 | 3.00E-03 |
| rh_medialorbitofrontal_part11_area | -0.035(0.059) | -0.055(0.053) | 3.06 | 0.028 |
| rh_medialorbitofrontal_part12_area | -0.046(0.062) | -0.066(0.048) | 3.12 | 0.025 |
| rh_medialorbitofrontal_part15_area | -0.007(0.059) | -0.030(0.051) | 3.58 | 0.008 |
| rh_middletemporal_part4_area | 0.011(0.057) | 0.029(0.055) | -3.13 | 0.024 |
| rh_middletemporal_part5_area | 0.063(0.043) | 0.076(0.033) | -2.83 | 0.046 |
| rh_middletemporal_part26_area | 0.047(0.055) | 0.064(0.039) | -3.37 | 0.014 |
| rh_parahippocampal_part3_area | 0.065(0.050) | 0.089(0.026) | -5.1 | 8.09E-05 |
| rh_parahippocampal_part4_area | 0.070(0.046) | 0.086(0.032) | -3.37 | 0.014 |
| rh_parahippocampal_part5_area | 0.061(0.059) | 0.093(0.024) | -6.18 | 3.73E-07 |
| rh_parahippocampal_part6_area | 0.066(0.058) | 0.087(0.034) | -3.91 | 3.00E-03 |
| rh_parahippocampal_part8_area | 0.087(0.043) | 0.100(0.021) | -3.21 | 0.021 |
| rh_pericalcarine_part1_area | -0.093(0.027) | -0.102(0.018) | 3.67 | 0.006 |
| rh_pericalcarine_part5_area | -0.086(0.034) | -0.095(0.025) | 2.96 | 0.035 |
| rh_postcentral_part35_area | -0.051(0.045) | -0.066(0.040) | 3.26 | 0.019 |
| rh_posteriorcingulate_part2_area | -0.095(0.015) | -0.101(0.016) | 3.33 | 0.015 |
| rh_posteriorcingulate_part3_area | 0.046(0.061) | 0.020(0.064) | 3.58 | 0.008 |
| rh_precentral_part7_area | -0.053(0.043) | -0.066(0.033) | 3.09 | 0.027 |
| rh_precuneus_part24_area | 0.029(0.063) | 0.049(0.053) | -3.32 | 0.016 |
| rh_rostralanteriorcingulate_part1_area | 0.031(0.061) | 0.002(0.061) | 4.13 | 2.00E-03 |
| rh_rostralanteriorcingulate_part4_area | 0.041(0.065) | 0.007(0.068) | 3.88 | 3.00E-03 |
| rh_rostralanteriorcingulate_part5_area | 0.028(0.051) | 0.004(0.052) | 4.15 | 2.00E-03 |
| rh_rostralmiddlefrontal_part2_area | 0.023(0.059) | 0.004(0.060) | 2.82 | 0.046 |
| rh_rostralmiddlefrontal_part7_area | -0.039(0.049) | -0.056(0.047) | 2.86 | 0.043 |
| rh_rostralmiddlefrontal_part11_area | -0.042(0.053) | -0.059(0.051) | 2.86 | 0.043 |
| rh_rostralmiddlefrontal_part22_area | -0.029(0.066) | -0.05(0.054) | 3.08 | 0.027 |
| rh_rostralmiddlefrontal_part23_area | -0.033(0.052) | -0.054(0.044) | 3.98 | 2.00E-03 |
| rh_rostralmiddlefrontal_part28_area | -0.041(0.047) | -0.060(0.045) | 3.97 | 3.00E-03 |
| rh_rostralmiddlefrontal_part40_area | -0.016(0.061) | -0.038(0.056) | 2.89 | 0.04 |
| rh_rostralmiddlefrontal_part46_area | -0.026(0.062) | -0.047(0.057) | 3.15 | 0.024 |
| rh_rostralmiddlefrontal_part49_area | -0.023(0.060) | -0.040(0.053) | 2.93 | 0.037 |
| rh_superiorfrontal_part2_area | -0.018(0.060) | -0.047(0.049) | 4.29 | 1.00E-03 |
| rh_superiorfrontal_part4_area | -0.009(0.061) | -0.031(0.062) | 3.38 | 0.014 |
| rh_superiorfrontal_part6_area | -0.012(0.063) | -0.030(0.055) | 3.00 | 0.032 |
| rh_superiorfrontal_part42_area | 0.027(0.061) | 0.006(0.065) | 2.90 | 0.039 |
| rh_superiortemporal_part1_area | 0.034(0.057) | 0.054(0.045) | -3.43 | 0.013 |
| rh_superiortemporal_part6_area | 0.048(0.054) | 0.070(0.044) | -3.83 | 4.00E-03 |
| rh_superiortemporal_part8_area | 0.055(0.046) | 0.079(0.035) | -4.75 | 2.51E-04 |
| rh_superiortemporal_part20_area | 0.052(0.042) | 0.072(0.032) | -4.00 | 2.00E-03 |
| rh_superiortemporal_part24_area | 0.033(0.037) | 0.061(0.032) | -6.72 | 2.94E-08 |
| rh_superiortemporal_part32_area | 0.022(0.046) | 0.047(0.042) | -4.42 | 1.00E-03 |
| rh_frontalpole_part2_area | -0.003(0.057) | -0.04(0.048) | 6.26 | 2.90E-07 |
| rh_temporalpole_part1_area | 0.021(0.057) | 0.051(0.039) | -5.00 | 1.18E-04 |
| rh_temporalpole_part3_area | 0.073(0.056) | 0.095(0.028) | -4.35 | 1.00E-03 |
| rh_temporalpole_part4_area | 0.040(0.044) | 0.062(0.033) | -4.71 | 2.89E-04 |

## Supplementary Table 3. The differences in the first MSN gradient in each functional network

| Network | AD | NCs | t-statistic | *p_FDR_* |
| --- | --- | --- | --- | --- |
| VIS | -0.038(0.008) | -0.041(0.007) | 4.28 | 1.13E-04 |
| SOM | 0.009(0.011) | 0.009(0.009) | 1.04 | 0.35 |
| DAN | -0.006(0.009) | -0.003(0.008) | -4.23 | 1.13E-04 |
| SAL | 0.024(0.007) | 0.022(0.007) | 3.05 | 4.36E-03 |
| LIM | 0.012(0.012) | 0.016(0.010) | -3.13 | 4.36E-03 |
| FPN | -0.006(0.008) | -0.007(0.007) | 0.75 | 6.00E-01 |
| DMN | 0.015(0.005) | 0.016(0.005) | -2.66 | 0.01 |

## Supplementary Table 4. The differences in the first MSN gradient in each cytoarchitectural class

| Cytoarchitecture class | AD | NCs | t-statistic | *p_FDR_* |
| --- | --- | --- | --- | --- |
| Prim motor | 0.028(0.018) | 0.029(0.015) | -0.30 | 0.79 |
| Asso1 | 0.009(0.004) | 0.012(0.004) | -6.82 | 1.89E-10 |
| Asso2 | 0.002(0.006) | 0.002(0.005) | -1.06 | 0.51 |
| Sec sens | -0.034(0.009) | -0.041(0.007) | 7.94 | 3.26E-13 |
| Prim sens | -0.032(0.010) | -0.035(0.008) | 3.74 | 5.18E-04 |
| Limbic | 0.013(0.014) | 0.013(0.013) | -0.27 | 0.79 |
| Insula | 0.052(0.015) | 0.050(0.017) | 0.90 | 0.52 |
|  |  |  |  |  |

## Supplementary Table 5. The differences in the first MSN gradient distribution

|  | statistic | *p* |
| --- | --- | --- |
| Global | 0.02 | 3.94E-52 |
| Prim motor | 0.02 | 0.022 |
| Asso1 | 0.03 | 2.00E-34 |
| Asso2 | 0.01 | 2.69E-05 |
| Sec sens | 0.07 | 5.07E-77 |
| Prim sens | 0.05 | 3.71E-21 |
| Limbic | 0.03 | 5.43E-05 |
| Insula | 0.06 | 1.29E-08 |

## Supplementary Table 6. Prediction of cognitive scores in AD

|  | cognitive scores | *r* | *p_perm_* |
| --- | --- | --- | --- |
| The first MSN gradient | MMSE | 0.15 | 0.06 |
|  | ADNI_MEM | 0.33 | <0.001 |
|  | ADNI_EF | 0.22 | 0.01 |
|  | ADNI_LAN | 0.33 | 0.001 |
|  | ADNI_VS | 0.42 | <0.001 |
| Regional MSN value | MMSE | -0.04 | 0.69 |
|  | ADNI_MEM | 0.12 | 0.10 |
|  | ADNI_EF | 0.18 | 0.03 |
|  | ADNI_LAN | 0.22 | 0.01 |
|  | ADNI_VS | 0.22 | 0.01 |

# Supplementary Figures


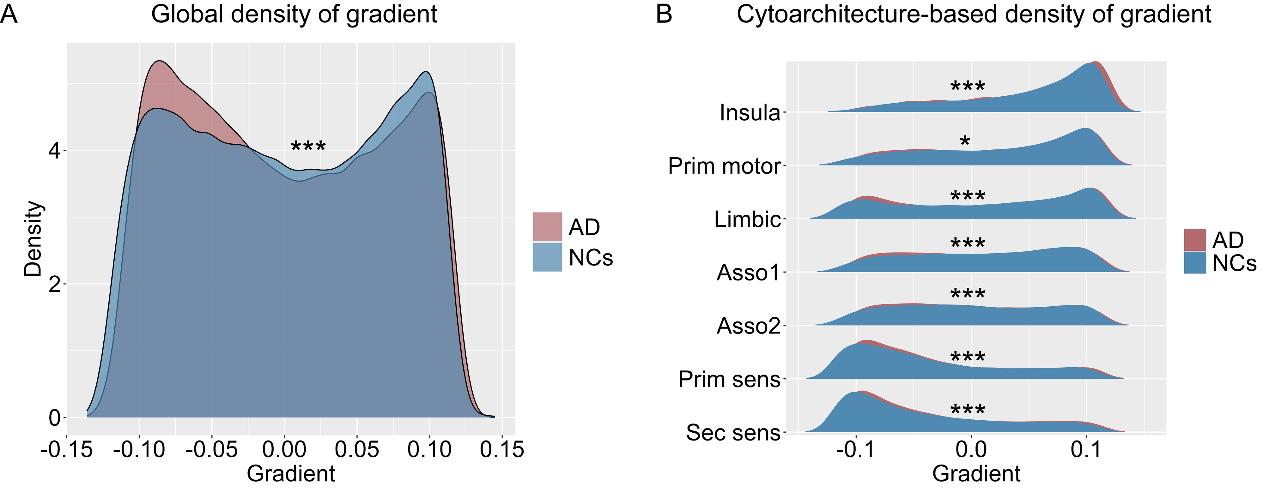


## Supplementary Figure 1 Distribution of the first MSN gradient in AD and NCs

A: Global distribution of the first MSN gradient in AD and NCs. B: Distribution of the first MSN gradient in AD and NCs based on Von Economo classes. *: *p* < 0.05, ***: *p* < 0.001.


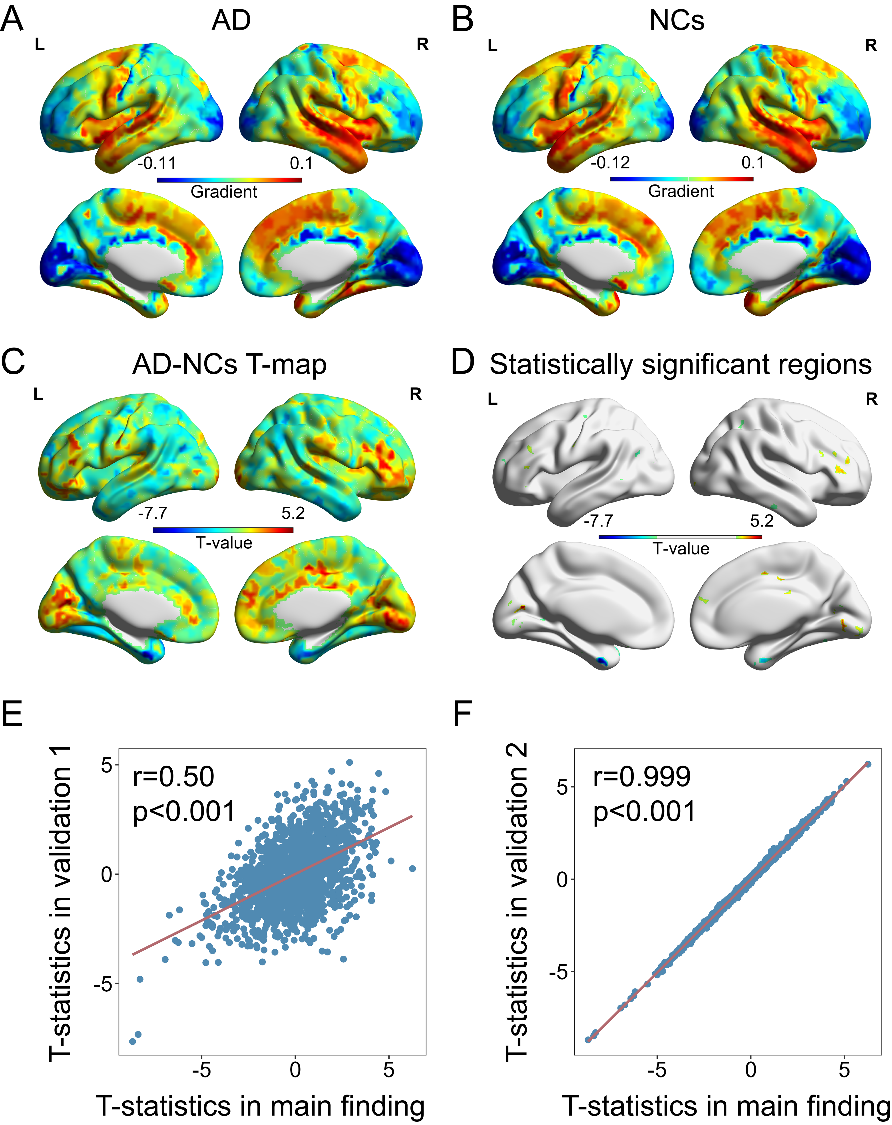


## Supplementary Figure 2 Results of the robustness analysis

A and B: the first MSN gradient mapping for ADs and NCs obtained in the validation set. C: t-statistic mapping of the results of intergroup comparisons at the level of brain regions of the first MSN gradient in the validation set. D: Brain regions with significant differences in the first MSN gradient for ADs and NCs in the validation set. E and F: Robustness of the results of intergroup comparisons at the level of brain regions of the first MSN gradient. validation 1: validation in the validation set, validation 2: TIV was used as a covariate when making comparisons of the first MSN gradient at the level of brain regions. In C and D, cool colors indicate regions with decreased first MSN gradients in AD compared to NCs, and warm colors indicate regions with increased first MSN gradients in AD compared to NCs.

# References

Arnatkeviciute, A., Fulcher, B. D., & Fornito, A. (2019). A practical guide to linking brain-wide gene expression and neuroimaging data. *Neuroimage, 189*, 353-367. doi:10.1016/j.neuroimage.2019.01.011

Hawrylycz, M. J., Lein, E. S., Guillozet-Bongaarts, A. L., Shen, E. H., Ng, L., Miller, J. A., . . . Jones, A. R. (2012). An anatomically comprehensive atlas of the adult human brain transcriptome. *Nature, 489*(7416), 391-399. doi:10.1038/nature11405

Markello, R. D., Arnatkeviciute, A., Poline, J. B., Fulcher, B. D., Fornito, A., & Misic, B. (2021). Standardizing workflows in imaging transcriptomics with the abagen toolbox. *Elife, 10*. doi:10.7554/eLife.72129

Rubinov, M., & Sporns, O. (2010). Complex network measures of brain connectivity: uses and interpretations. *Neuroimage, 52*(3), 1059-1069. doi:10.1016/j.neuroimage.2009.10.003

Shen, C., Rolls, E. T., Cheng, W., Kang, J. J., Dong, G. Y., Xie, C., . . . Feng, J. F. (2022). Associations of Social Isolation and Loneliness With Later Dementia. *Neurology, 99*(2), E164-E175. doi:10.1212/wnl.0000000000200583

Xia, M., Liu, J., Mechelli, A., Sun, X., Ma, Q., Wang, X., . . . He, Y. (2022). Connectome gradient dysfunction in major depression and its association with gene expression profiles and treatment outcomes. *Molecular Psychiatry, 27*(3), 1384-1393. doi:10.1038/s41380-022-01519-5

Zheng, C. C., Xiao, X. X., Zhao, W., Yang, Z. Y., & Guo, S. X. (2024). Functional brain network controllability dysfunction in Alzheimer's disease and its relationship with cognition and gene expression profiling. *Journal of Neural Engineering, 21*(2). doi:10.1088/1741-2552/ad357e

Zheng, C. C., Zhao, W., Yang, Z. Y., & Guo, S. X. (2024). Functional connectome hierarchy dysfunction in Alzheimer's disease and its relationship with cognition and gene expression profiling. *Journal of Neuroscience Research, 102*(1). doi:10.1002/jnr.25280

1. † Data used in preparation of this article were obtained from the Alzheimer's Disease Neuroimaging Initiative (ADNI) database (adni.loni.usc.edu). As such, the investigators within the ADNI contributed to the design and implementation of the ADNI and/or provided data but did not participate in the analysis or writing of this report. A complete listing of ADNI investigators can be found at: https://adni.loni.usc.edu/wp-content/uploads/how_to_apply/ADNI_Acknowledgement_List.pdf. [↑](#footnote-ref-1)
